# Characteristics of Adolescents Admitted with Acute Alcohol Intoxication: A Retrospective Multicentre Study in Antwerp, Belgium, in the Period 2015–2021

**DOI:** 10.3390/children10081378

**Published:** 2023-08-12

**Authors:** Hanna van Roozendaal, Stijn Verhulst, Inge Glazemakers, Frederic De Meulder, Ann Vander Auwera, Anna Bael, Emmi Van Damme, Ilse Vlemincx, Jozef De Dooy, Nico van der Lely, Guido Van Hal

**Affiliations:** 1 Faculty of Medicine and Health Sciences, University of Antwerp, 2610 Wilrijk, Belgium; inge.glazemakers@uantwerpen.be (I.G.); anna.bael@zna.be (A.B.); jozef.dedooy@uza.be (J.D.D.); n.vanderlely@rdgg.nl (N.v.d.L.); guido.vanhal@uantwerpen.be (G.V.H.); 2 Department of Paediatrics, Antwerp University Hospital, 2650 Edegem, Belgium; 3 University Centre for Child and Adolescent Psychiatry (ZNA-UKJA), 2020 Antwerp, Belgium; 4 Department of Paediatrics, GasthuisZusters Antwerpen (GZA), 2018 Antwerp, Belgium; frederic.demeulder@gza.be (F.D.M.); ann.vanderauwera@gza.be (A.V.A.); 5 Department of Paediatrics, Hospital Network Antwerp (ZNA), 2020 Antwerp, Belgium; emmi.vandamme@zna.be; 6 Department of Paediatrics, General Hospital (AZ) Monica, 2100 Antwerp, Belgium; ilse.vlemincx@azmonica.be; 7 Department of Paediatrics, Reinier de Graaf Hospital, 2625 AD Delft, The Netherlands

**Keywords:** adolescents, minors, alcohol intoxication, substance use, population characteristics, Belgium

## Abstract

Binge drinking among adolescents is common in Belgium, posing a risk of serious health consequences. Until today, only estimations of the prevalence of acute alcohol intoxication (AAI) in adolescents have been made. Research into potential risk factors has not yet been conducted in Belgium. Therefore, this study aims to gain more insight into the prevalence, medical characteristics and potential risk factors of AAI among adolescents. A retrospective multicentre chart study was performed on adolescents aged 10–17 years with AAI in Antwerp, Belgium (2015–2021). Patient’s demographics, medical characteristics and information regarding the context of the AAI were collected from medical charts. Over the study period, a total of 1016 patients were admitted with AAI in Antwerp, having a median age of 16.6 years old, a median blood alcohol concentration of 1.95 g/L and combined drug use in 10% of cases. These findings did not significantly change over the study period. Multiple linear regression analysis indicated that after correcting for covariates, higher age, no combined drug use and decreased consciousness at admission were associated with more severe AAI cases (higher blood alcohol concentration). This study shows that AAI is prevalent among Belgian adolescents, and better targeted preventive measures and policies are needed. Our findings could be taken into account when developing preventive measures. However, data addressing the demographics and context of AAI were mostly missing. Therefore, prospective research is required to further investigate potential risk factors associated with AAI.

## 1. Introduction

Alcohol abuse has a major public health impact. Indeed, 5.3% of all global deaths in 2016 were attributable to alcohol [1]. More specifically, adolescents are susceptible to the negative health impacts of alcohol, since the pre-frontal cortex of their brain is still in development, resulting in lower impulse control and increased sensation-seeking behaviour [2,3]. As a result, risky alcohol use is common in adolescents [4], such as binge drinking, i.e., drinking at least four (females) or six (males) alcoholic beverages in two hours [5], and acute alcohol intoxication (AAI), which is a harmful clinical condition that occurs following the ingestion of a large amount of alcohol that often needs medical management [6]. Also, in Belgium, excessive use of alcohol by adolescents is common, which has been illustrated by recent nationally and internationally conducted health surveys. Firstly, although the minimum age limit for the use of alcohol in Belgium is 16, more than half of Flemish 15-year-olds drink at least once a month, compared to 37% in Europe and Canada [7]. Secondly, 21% of the Flemish 15- and 16-year-olds and 37% of the Flemish 17- and 18-year-olds binge drink at least once a month [8]. Furthermore, 10.4% of Belgian youth (15-to-24-year-olds) drink more than six alcoholic beverages on one occasion every week [9]. These data, along with the fact that emergency doctors in Belgium have recently alerted society to the increasing number of adolescents admitted to hospital due to AAI, emphasize the severity of (binge) drinking among adolescents in Belgium. Therefore, Belgian adolescents are at risk of serious health consequences related to excessive alcohol use. In the short term, problematic alcohol use in adolescents is associated with violence, accidents, sexual victimisation, hypothermia, coma and death [4,10]. Furthermore, it increases the risk of brain damage [11], alcohol dependency or addiction [12] and alcohol-attributable cancers in the long term [13,14].

As suggested by Shield et al., cost-effective local and national policy measures that can reduce alcohol use and the resulting burden of alcohol-related disease are needed [1]. This observation is also in line with the European framework for action on alcohol developed by the World Health Organisation [15]. To develop cost-effective preventive measures to combat problematic alcohol use in adolescents, an understanding of the population characteristics of this group of adolescents is necessary. These traits are demonstrated by, amongst others, the Dutch research group of Van der Lely et al., which has shown that enlarged insight into the prevalence and characteristics of AAI in adolescents in the Netherlands has resulted in effective national policy measures [8]. However, in Belgium, although considerable research has been devoted to addressing general drinking behaviour in adolescents using self-report surveys [8,9], less attention has been paid to addressing AAI in this population. Until now, only estimations of the numbers of adolescents admitted to hospitals in Belgium due to AAI have been made, which were based on approximations of health insurance data [16]. Furthermore, research regarding the context of AAI or potential risk factors of AAI has not yet been conducted in Belgium. Therefore, further research regarding AAI in adolescents in Belgium is needed to ensure that more suitable preventive measures can be developed to reduce problematic alcohol use in adolescents.

Therefore, the present study aims to offer insight into the prevalence, (medical) characteristics and potential risk factors of AAI among adolescents in the region of Antwerp, Belgium, over a 7-year period (2015–2021).

## 2. Materials and Methods

### 2.1. Study Design

A retrospective observational study was performed in adolescents who experienced alcohol-related emergency department (ED) admissions in the eight hospitals with an ED in Antwerp, Belgium, from 2015 until 2021. The city of Antwerp has around 530,000 inhabitants, of which 50,000 are 10–17 years old [17]. In total, there are 15 hospitals in the city of Antwerp, of which 8 have an ED. The participating hospitals were six hospitals managed by the Hospital Network Antwerp (GZA-ZNA) and two hospitals managed by the Helix network, including the Antwerp University Hospital (UZA).

### 2.2. Inclusion and Exclusion Criteria

Patients were identified by clinical biologists working at the participating hospitals, who selected all patients aged 10–17 years old who had a positive blood alcohol concentration (BAC, >0.03 or >0.1 g/L, depending on the laboratory) and were admitted between January 2015 and December 2021. The minimum age of 10 years old was determined to focus on adolescents who were old enough to intentionally consume alcohol and reduce the likelihood of including patients with accidental ingestion. This minimum age is in line with national-level studies from other countries [18,19,20]. Identification of patients via laboratory results was complemented using ED triage logs, namely ‘seemingly drunk’ and ‘intoxication’ (UZA), and screening of hospital charts via screening software, making use of the search terms ‘intoxication’, ‘alcohol’, ‘ethanol’ and ‘drunkenness’ (GZA), taking into account the same 7-year period. All identified patients’ medical records were reviewed by the researchers. Subsequently, all 10–17-year-old patients with a positive BAC and/or clinically diagnosed alcohol intoxication by the emergency doctor were included in this study. Exclusion criteria covered patients who, based on their history, did not drink alcohol and patients with a negative BAC.

### 2.3. Data Collection

We reviewed all included patients’ medical charts in a standardised way to collect their demographics, medical characteristics and variables regarding the context of their admission. In this respect, demographic variables were mainly collected from the patient information part of the medical chart, including age (calculated using their date of birth and date of hospitalisation), sex, nationality and the first two numbers of their postal code. Furthermore, correspondence with the treating physician and/or nurse, assessment tools and/or scales filled in by the nurse and laboratory results were used for the collection of medical characteristics of each patient’s hospitalisation. In this regard, multiple variables have been collected: date and time of admission and discharge, the reason for admittance, the Glasgow Coma Scale (GCS, i.e., a scale used to objectively describe the extent of impaired consciousness in patients, with 15 being the best response and 3 being the lowest response, meaning that a patient was totally unresponsive), BAC (in g/L), urine drug screening (whether performed and result when performed), admission to the intensive care unit (ICU), consultation with other healthcare workers and referral details (if the patient was referred to other internal or external healthcare workers). Finally, variables regarding the context of the admission were collected, when available, from the medical charts. In this respect, the type of alcohol product and number of glasses consumed were abstracted from the correspondence of the treating physician and/or nurse, and the type of transport to the hospital and the involvement of the police were abstracted from either this correspondence or scanned documents.

Data collection was performed by two of the team’s researchers, who randomly cross-checked each other’s findings to improve reliability. 

### 2.4. Statistical Analysis

Descriptive statistics were expressed as proportions for categorical variables and as medians [interquartile range (IQR)] for continuous variables because of a non-normal distribution, which was checked by assessing histograms and Q-Q plots and performing Kolmogorov–Smirnov tests (*p* < 0.001 for all continuous variables in this study). The annual hospitalisation rate was calculated by dividing the number of hospitalisations recorded in each year by the corresponding number of inhabitants as of January each year [17] and expressed per 10,000 inhabitants. Pearson’s chi-squared tests were used to analyse associations between categorical variables addressing demographics (age and sex) and medical characteristics (annual hospitalisation rate, reason for admission, GCS, time of admission, results of urine drug screening, admission at ICU, transport and police involvement). To assess the univariable association between categorical variables and median BAC, a Mann–Whitney U test was used for variables with two categories (sex, day of admittance and type of transport), and a Kruskal–Wallis test was used for variables with more than two categories (combined drug use, reason for admittance and location of drinking). To assess the univariable association between age (a non-normal distributed continuous variable) and median BAC, Spearman’s correlation coefficient was performed. Statistically significant variables from the univariable analyses were included in a multiple linear regression model to determine the association between BAC and multiple AAI characteristics.

The significance level for all statistical tests was set at α = 0.05. Statistical analyses were performed using IBM SPSS Statistics for Windows, Version 28.0 (Armonk, NY, USA: IBM Corp).

The study received approval from the medical ethics committees of all participating hospitals (central project ID 2021-0412).

## 3. Results

### 3.1. Hospitalisation Rate

**Figure 1:**
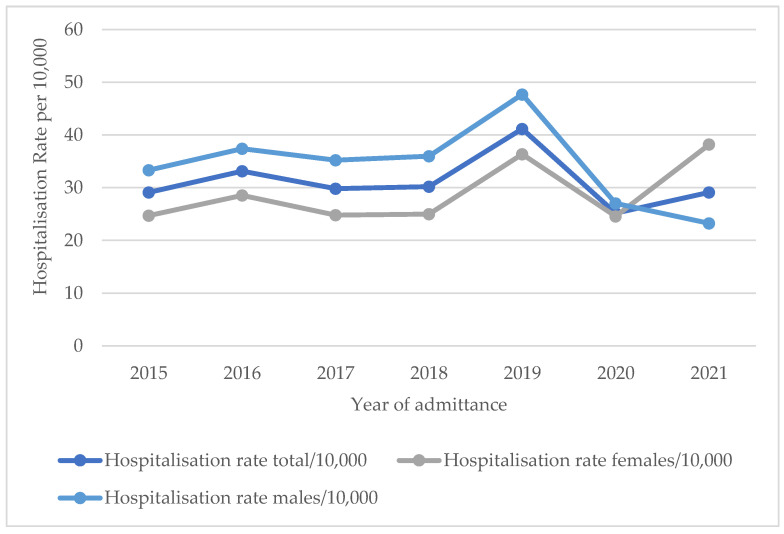
Hospitalisation rates of alcohol-related admissions per 10,000 adolescents aged 10–17 years old in Antwerp in the period 2015–2021.

Over the study period, a total of 1032 hospital admissions related to alcohol occurred in 1016 patients aged 10–17 years old. This number of patients represents a mean annual hospitalisation rate of 31 per 10,000 10–17-year-old inhabitants in the city of Antwerp over the study period, with a significant increase in the annual hospitalisation rate occurring in 2019 (*p* < 0.001), though it stabilised in 2020, as shown in Figure 1.

The median age of these 1016 patients was 16.6 years old [15.6–17.4], and it did not change significantly over the study period (*p* = 0.1). However, the minimum age decreased from 12.6 years old in 2015 to 11.1 years old in 2021, as set out in Table 1.

**Figure 2:**
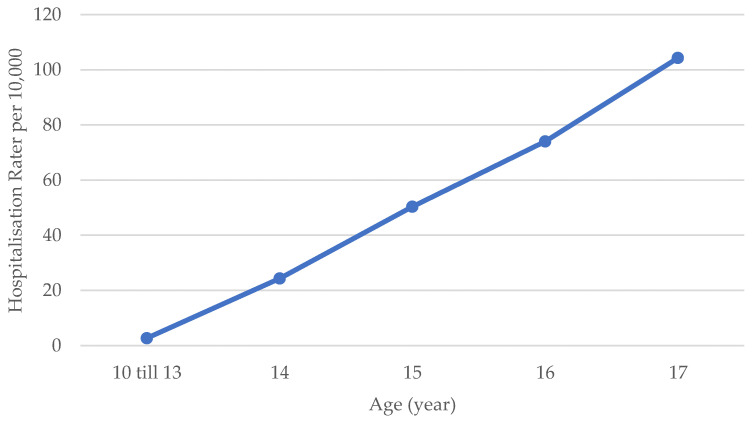
Mean hospitalisation rate of alcohol-related admissions per 10,000 corresponding adolescents in Antwerp in the period 2015–2021.

Furthermore, 33.4% of the patients were below the legal alcohol consumption age of 16 years old at the time of admission. Figure 2 shows the mean hospitalisation rate per 10,000 inhabitants per age group. The higher the age group, the higher the mean hospitalisation rate, with significant differences shown per age group (*p* < 0.001).

Overall, the admission rate of males was significantly higher (*p* = 0.006), with 56.3% of patients being males and 43.7% of patients being females and taking into account a ratio of 0.52 males to 0.48 females within the population of adolescents aged 10–17 years old in Antwerp [17]. However, up to the age of 14 years old, significantly more females were admitted (*p* = 0.02). At the ages of 15 and 16 years old, the admission rate was equal; at 17 years old, more males were admitted (*p* < 0.001). Interestingly, significantly more females than males were admitted to the hospital due to AAI in 2021, which is in contrast to the years 2015 through 2020 (*p* = 0.006), as illustrated in Figure 1.

### 3.2. General Characteristics

An overview of the medical characteristics of the 1016 patients who experienced alcohol-related ED admissions is provided in Table 2.

As shown in Table 2, urine drug screening was performed in 30.3% of cases (308/1016), of which 17.5% (54/308) were positive for cannabis, cocaine, amphetamines and/or benzodiazepines. Cannabis was the drug most often used in combination with alcohol, followed by amphetamines (including XTC/MDMA) and benzodiazepines. Although urine drug screening was performed significantly more frequently in females (33.8% of the females were screened versus 27.7% of the males, *p* = 0.04), no significant difference in drug use was seen between males and females (*p* = 0.14). Following the confirmation of drug use via urine drug screening, drug use was self-reported by a total of 46 patients, of whom 74% reported the use of cannabis. Nitrous oxide use could not be detected via urine drug screening; nevertheless, three patients reported the use of this substance. When combining urine drug screening and self-reported drug use, a total of 100 patients (9.8%) used (illegal) drugs in combination with alcohol. Overall, drug use was significantly more common in patients of an older age: the median age in patients with combined drug use was 16.6 [15.7–17.5], in contrast to 16.2 [15.1–17.1] in patients without combined drug use (*p* = 0.002).

In 900 patients (88.6%), the primary reason for admittance was shown on their medical chart. In half of these cases (50.3%), the primary reason was decreased consciousness. Furthermore, in 13.9% of cases, there was a (traffic) accident, which was significantly more frequently seen in males than in females (*p* = 0.009). Other reported primary reasons for admittance were syncope, vomiting, suicide attempts, aggression, inappropriate behaviour or concern among bystanders, which are combined into one category in Table 2. An overview of all patients with reduced consciousness was made, combining patients with decreased consciousness as the primary reason for admittance and/or patients with a result of 14 or lower on the GCS. As shown in Table 2, a total of 566 patients (55.7%) were considered to suffer from decreased consciousness. No significant differences in reduced consciousness were seen between males and females (*p* = 0.15) or between different age groups (*p* = 0.5).

Remarkably, most of the patients were admitted to the hospital during the evening and at night time (86.9%), with significantly more males presenting during the night and more females presenting during the evening and afternoon (*p* = 0.04). Furthermore, patients were admitted more frequently over the weekend (70.5%) than during the week (29.5%). When looking at fluctuations in admissions over a period of a year, a slight increase in AAI admissions was seen in the months June and December (n = 103 and n = 104, respectively, compared to a mean number of admissions of n = 85 per month). However, these fluctuations were not significant. In addition, most patients were discharged the following morning, with the median duration of admittance being 7.5 h [4–11.5].

To gain insights into the social impact and costs, data on transport and police involvement were abstracted from the medical charts when available. Table 2 shows that most patients (83.2%) were transported to the hospital via ambulance or ambulance with specialised care (in the presence of a specialised doctor and nurse). Furthermore, in 15.3% of cases, police involvement was reported. There were no significant sex or age differences related to the type of transport (*p* = 0.39 and *p* = 0.53, respectively) and police involvement (*p* = 0.24 and *p* = 0.62, respectively).

### 3.3. Recurrence Rate

Of the 1032 total admissions, 16 were recurrent admissions (1.6%), which occurred in 15 patients. These patients had a median age of 17.2 years old [16.4–17.7], which was older than the overall average age for the group of patients. Furthermore, they had a median BAC of 2.09 g/L [0.86–2.57], which was higher than that of the group of patients with only one admission.

### 3.4. Severeness of AAI

**Figure 3:**
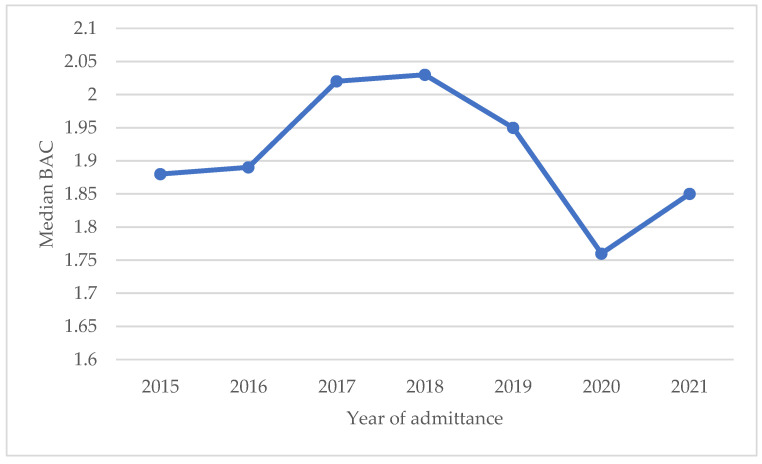
Trend in median blood alcohol concentration over time.

An important clinical characteristic of AAI is blood alcohol concentration. As shown in Table 2, median BAC was 1.95 g/L, having a significantly higher BAC in males than in females (*p* = 0.04). When analysing trends in BAC over the study period, a significant decrease in median BAC was observed in 2020 compared to other years (*p* = 0.03), as shown in Figure 3.

A multiple regression was run to investigate the influence of patient characteristics on BAC, which is a measure of the severity of AAI. Table 3 shows the median BACs of subgroups of available patient characteristics that could potentially affect BAC based on the literature [20,21,22], including the test results of the univariable associations.

The outcome of the multiple regression analysis is presented in Table 4. Age, combined drug use and the reason for admittance continued to be significantly associated with BAC. The R^2^ of the model was 28%, having F(5.839) = 66,282, *p* < 0.001 and a good model fit (all assumptions were met).

## 4. Discussion

This study is the first study to address the prevalence and medical characteristics of adolescents with AAI and factors associated with severe AAI in the region of Antwerp, Belgium, by retrospectively investigating patients’ medical records.

The results of this study show that, on average, 31 people per 10,000 10-to-17-year-olds were admitted to hospital with AAI in the city of Antwerp every year over the study period, which corresponds to 145 adolescents per year. Furthermore, this study found no decrease in the prevalence of AAI in adolescents over the study period. This finding is in line with estimations of Belgian health insurance invoice data [16] and the yearly Flemish pupil questionnaire [23]; the latter showed consistent patterns of binge drinking in adolescents from 2000 to 2019. However, our data showed a significant increase in prevalence in 2019, which levelled off in 2020. A possible hypothesis here could be that an increase in prevalence had initially started but subsequently weakened due to international COVID-19 restrictions. Accordingly, such a decrease in prevalence during the initial COVID-19 lockdown has been shown by Dutch and Italian researchers [24,25]. Further follow-up studies are needed to confirm this hypothesis.

When looking at characteristics of AAI in adolescents, our study showed a median age of 16.6 years old, with no significant change over the study period. This result is consistent with a 5-year cohort study conducted in Wales [18]. However, in the Netherlands, researchers found increases in the mean age in both 4- [26] and 10-year follow-up studies [27], which were probably due to the increase in the legal drinking age in 2014 [27]. Considering that AAI at 17 years increased significantly in our study and the legal drinking age in Belgium is still set at 16 years old, the impact of increasing the legal drinking age from 16 to 18 years old could have an important impact on increasing the median age of adolescents with AAI, with a lower risk of alcohol dependence later in life as a result [12,28,29]. In addition, consistent with previous studies [27,30,31,32,33], significantly more boys were admitted with AAI over the study period. However, up to the age of 14 years old, more girls with AAI were admitted. Accordingly, similar differences have been found in other European studies [18,27,30]. This finding may be explained by the theory that puberty, i.e., the phase in which adolescents show increased sensation-seeking behaviour and experimentation, starts earlier in girls than in boys [34]. Moreover, consistent with other studies [20,33,35], almost 10% of the patients combined alcohol with other drugs, of which cannabis was the most common choice. However, an underestimation of substance use should be considered, because only 30% of patients were screened for combined drug use and there were limitations to urine drug screening, namely the failure to detect novel psychoactive drugs. Conversely, combined cannabis use is potentially overestimated due to the long detection time of cannabis metabolites in urine. Nevertheless, increasing the amount of urine drug screening at the ED could help to determine the severity of intoxication and, therefore, improve treatment and counselling of patients and their families. In addition, BAC screening showed a stable median BAC of 1.95 g/L, which was similar to those of previous studies from Slovenia [36] and the Netherlands [20,37]. This high BAC at admittance might even be an underestimation due to the delay between drinking alcohol and undergoing blood screening at the ED. Factors associated with more severe AAI in adolescents were investigated using multiple linear regression analysis on BAC. Roughly 28% of a higher BAC in intoxicated adolescents could be explained based on higher age, no combined drug use and reduced consciousness. Higher BAC in relation to age may be explained by a decreased sensitivity to alcohol, which is the result of more frequent alcohol use [20]. Accordingly, this observation could be taken into account when developing preventive measures, such as targeted campaigns or increasing the legal drinking age. When successfully increasing the age of initial alcohol consumption in adolescents, decreased sensitivity to alcohol will develop at an older age, which will probably lead to a lower BAC in adolescents with AAI, even at an older age. In contrast to our data, a significant association between sex and BAC has been shown in the Netherlands [20]. In our data, this lack of association seems to be explained based on other factors, mainly age; girls were admitted at a significantly younger age, and age is associated with a lower BAC. Other studies also showed an association between drinking location, the age of initial alcohol consumption and educational level and BAC [20]; however, in our study, no or not enough data were available in this regard.

The results of this study should be considered in the context of certain limitations of the design. Firstly, different inclusion methods have been used, according to the methods available in participating hospitals. In this regard, a different BAC sensitivity was seen in different hospital laboratories (0.1 and 0.03 g/L), and only in some hospitals was screening of triage logs and hospital charts using screening software possible. This issue may have led to selection bias. Secondly, the recurrence rate of 1.6% might be an underestimation due to the anonymity of patients between the various hospitals. Previous studies presented higher recurrence rates in this respect [18,33,38]. Therefore, prospective studies in the future, for instance, a national registration system, could correct for this issue. Thirdly, as our data were derived from hospital charts, we were dependent on the notes of healthcare providers. This issue might have caused information bias due to missing data related to nationality, religion, education level and variables regarding the context of the alcohol intoxication. Furthermore, information bias may have been introduced based on the choice of the health care provider to report additional data. For instance, a doctor would probably report more information regarding the context of AAI or be more likely to perform a urine drug screening when the clinical condition is considered to be more severe. Not testing for drug use, in this regard, may give a higher chance of registering no combined drug use in our study. Finally, we hypothesise that the patients included in our study represent only a part of the total prevalence in Antwerp. For instance, certain adolescents with AAI may visit a general practitioner instead of the ED or stay at home under the observation of a parent. Unreported cases were missed in our study, which might have resulted in an underestimation of the total prevalence of AAI in adolescents.

Nevertheless, despite the limitations mentioned above, the strengths of the study include the total population-based approach over 7 years and large sample size (n = 1016), which ensured thorough analysis. Furthermore, a nearly complete overview of patients with AAI admitted to the hospital has been made by avoiding using error-prone ICD coding for the selection of patients.

The results of this study allow us to conclude that AAI in adolescents in Antwerp is a severe and consistent problem. Although frequent alcohol use in Flemish adolescents is decreasing based on self-reported questionnaires [23], our study shows that the prevalence of AAI is not reducing, which makes it a serious and relevant public health problem. In this regard, the results of this study might be used to develop better-targeted preventive measures and policies. However, to develop suitable public health interventions, further research is imperative to improve our understanding of specific risk and protective factors of AAI in adolescents. This focus may be established by developing a prospective registration system for healthcare providers throughout Belgium, where complete and consistent data could be assembled. Moreover, our study showed that the majority of patients do not receive additional care after emergency admission (73%). However, as stated in previous research [20,30,38], family treatment and motivational enhancement therapy following initial treatment in emergency rooms is recommended. Thus, to decrease alcohol and substance use in our population, lower the risk of recurrence and generate prospectively obtained data for the development of targeted public health interventions, an outpatient clinic to treat adolescents with AAI in Antwerp will be set up by the researchers involved in this study, at which screening, parent support and motivation interviewing functions will be integrated.

## Figures and Tables

**Table 1:** Median, minimum and maximum ages of adolescents admitted with acute alcohol intoxication (AAI) per year.

Year of Admittance	Median Age (Years) [IQR]	Minimum Age	Maximum Age
2015	16.8 [15.9–17.5]	12.6	17.9
2016	16.8 [15.8–17.4]	13.1	17.9
2017	16.8 [15.4–17.5]	13.4	17.9
2018	16.7 [15.5–17.4]	13.0	17.9
2019	16.5 [15.5–17.4]	11.7	17.9
2020	16.3 [15.3–17.2]	11.1	17.9
2021	16.5 [15.4–17.2]	11.2	17.9

**Table 2:** Medical characteristics of 1016 patients aged 10–17 years old who experienced alcohol-related emergency admissions in Antwerp between 2015 and 2021.

Characteristic	Total Group of Patients
	*n* = 1016
**Sex, n (%)**	
Male	572 (56.3)
Female	444 (43.7)
**Age (years)**	
Median [IQR]	16.6 [15.6–17.4]
Range	11.1–17.9
**Year of admission, n (%)**	
2015	126 (12.4)
2016	146 (14.4)
2017	135 (13.3)
2018	141 (13.9)
2019	197 (19.4)
2020	124 (12.2)
2021	147 (14.5)
**Blood alcohol concentration (BAC, g/L), (n = 841)**	
Median [IQR]	1.95 [1.40–2.34]
Range	0.03–4.09
**Urine drug screening (n = 308), n(%)**	
Negative	254 (82.5)
Positive for cannabis	35 (11.4)
Positive for cocaine	3 (1.0)
Positive for XTC/amphetamines	17 (5.5)
Positive for benzodiazepines	12 (3.9)
**Glasgow Coma Scale (GCS, points), (n = 839)**	
Median [IQR]	14 [12–15]
Range	3–15
3–14 points, n(%)	431 (51.4)
15 points, n(%)	408 (48.6)
**Duration of stay (hours) (n = 1015)**	
Median [IQR]	7.5 [4–11.5]
Range	<0.5–549
<24 h, n(%)	906 (89.3)
**Reason for admittance (900), n (%)**	
Reduced consciousness	453 (50.3)
(Traffic) accident	125 (13.9)
Other (somatic/psychiatric)	322 (35.8)
**Decreased consciousness, n (%)**	
GCS ≤ 14 and/or reason for admittance = reduced consciousness	566 (55.7)
**Time of admittance, n (%)**	
Night (0:00–6:00 a.m.)	555 (54.6)
Morning (6:00 a.m.–12:00)	45 (4.4)
Afternoon (12:00–6:00 p.m.)	89 (8.8)
Evening (6:00 p.m.–0:00)	327 (32.3)
**Day of admittance, n (%)**	
Monday–Thursday	300 (29.5)
Friday–Sunday	716 (70.5)
**Intensive care unit (ICU), n (%)**	
Admittance at ICU	56 (5.5)
**Transport to emergency department (ED, n = 824), n(%)**	
Ambulance	539 (65.4)
Ambulance with specialised care	147 (17.8)
Police car	9 (1.1)
Attended by themselves	12 (1.5)
Attended with family or friends	117 (14.2)
**Police involvement, n (%)**	
	155 (15.3)
**Referral after admittance (n = 697), n (%)**	
None	509 (73)
Paediatrician	25 (3.6)
General practitioner	48 (6.9)
Psychosocial assistance	32 (4.6)
Child and adolescent psychiatry	63 (9.0)
Addiction care	7 (1.0)

**Table 3:** Median blood alcohol concentration per subgroup, including results of univariate analyses.

Characteristic	n (% of total)	Median BAC (g/L) [IQR]	Univariate *p*-Value
**Total**	841 (82.8)	1.95 [1.4–2.3]	
**Age (year)**			<0.001 *
10–13	44 (93.6)	1.39 [0.06–1.93]	
14	84 (86.6)	1.79 [1.24–2.2]	
15	158 (81.0)	1.86 [1.38–2.2]	
16	228 (80.9)	1.99 [1.50–2.36]	
17	327 (82.8)	2.03 [1.56–2.44]	
**Sex**			0.03 *
Male	479 (83.7)	1.99 [1.48–2.37]	
Female	362 (81.5)	1.85 [1.33–2.30]	
**Combined drugs use**			<0.001 *
No combined drug use	233 (95.1)	1.91 [1.43–2.39]	
Unknown	523 (78.3)	1.99 [1.48–2.37]	
Combined drug use	84 (82.4)	1.60 [0.97–2.03]	
**Reason for admittance**			<0.001 *
(Traffic) accident	108 (86.4)	0.78 [0.06–2.14]	
Other (somatic/psychiatric)	249 (77.3)	1.60 [0.85–2.05]	
Unknown	95 (81.9)	2.01 [1.65–2.37]	
Reduced consciousness	389 (85.9)	2.13 [1.77–2.48]	
**Type of transport**			0.28
Without ambulance	295 (89.4)	1.88 [1.31–2.30]	
Ambulance	546 (79.6)	1.98 [1.46–2.37]	
**Location of drinking**			0.08
At home (or at another person’s home)	66 (88.0)	1.94 [1.36–2.38]	
In public	60 (85.7)	2.12 [1.59–2.37]	
At school/work	19 (76.0)	2.11 [1.58–2.77]	
Youth organisation/sports club	33 (82.5)	2.26 [1.79–2.51]	
Commercial/event	152 (84.4)	2.13 [1.78–2.49]	
**Day of admittance**			0.001 *
Monday-Thursday	260 (86.7)	1.81 [1.03–2.27]	
Friday-Sunday	581 (81.1)	1.99 [1.50–2.37]	

* Statistically significant.

**Table 4:** Results of multiple regression analysis of patient characteristics with outcome blood alcohol concentration (g/L).

Variable	B Unstandardised Regression Coefficient	95% Confidence Interval	Multiple Regression *p*-Value
Age	0.145	[0.107, 0.183]	<0.001 *
Sex	−0.064	[−0.161, 0.034]	0.2
Combined drug use	−0.236	[−0.401, −0.072]	0.005 *
Reason for admittance	0.683	[0.597, 0.769]	<0.001 *
Day of admittance	0.081	[−0.24, 0.186]	0.13

* Statistically significant.

## Data Availability

The data that support the findings of this study can be requested from the corresponding author. When the authors are in mutual agreement, the data will be provided.
